# Suicide prevention program on suicidal behaviors and mental wellbeing among school aged adolescents: a scoping review

**DOI:** 10.3389/fpubh.2025.1506321

**Published:** 2025-05-02

**Authors:** Yasmin Nadeem Parpio, Rozina Nuruddin, Tazeen Saeed Ali, Nuruddin Mohammad, Uzma Rahim Khan, Salman Shahzad, Murad Musa Khan, Mehreen Aslam

**Affiliations:** ^1^Aga Khan University School of Nursing and Midwifery, Karachi, Pakistan; ^2^Aga Khan University, Aga Khan University Medical College, Karachi, Pakistan; ^3^Institute of Clinical Psychology, Karachi, Pakistan; ^4^Aga Khan University, Aga Khan University Brain and Mind Insitute, Karachi, Pakistan; ^5^National University of Medical Sciences (NUMS), Punjab, Pakistan

**Keywords:** prevention, strategies, intervention, measures, mental wellbeing, adolescent, suicide, suicidal behaviors

## Abstract

**Introduction:**

Suicide is a significant public health concern among school-aged adolescents. This scoping review aimed to synthesize evidence-based literature on suicide prevention interventions designed to enhance mental wellbeing and reduce suicidal behavior in this population.

**Methods:**

Following the PRISMA-ScR guidelines and Arksey and O’ Malley’ s five-phase framework, a systematic search was conducted across five databases (PubMed, Psych Info, Google Scholar, Science Direct, EBSCO, and ProQuest) to identify relevant studies meeting the inclusion criteria. Key findings from 37 peer-reviewed papers on effective strategies and interventions were synthesized.

**Results:**

The reviewed studies encompassed diverse interventions, including school-based programs like Signs of Suicide (SOS) student training and digital platforms, as well as therapeutic and family-based approaches for school-age adolescents. Outcomes consistently demonstrated significant improvements in mental wellbeing, self-efficacy, and help-seeking behaviors. Furthermore, reductions in suicidal ideation and self-harm were observed across these interventions.

**Discussion:**

The findings highlight the effectiveness of various suicide prevention interventions for school-aged adolescents. Future research should focus on integrating diverse and culturally sensitive school-based mental health services and risk screening programs, adapting interventions to align with specific cultural norms and values, particularly in low- and middle-income countries where research is lacking. Enhancing access to support and implementing resilience-building initiatives at the primary school level are crucial for empowering adolescents with essential help-seeking skills and resources, fostering a supportive environment for mental wellbeing and suicide prevention. A limitation of this review is the exclusion of non-peer-reviewed literature. Policymakers and school administrators should consider these findings when developing and implementing suicide prevention programs.

**Systematic review registration:**

This review is registered in open science framework in 2022 ID: https://doi.org/10.17605/OSF.IO/6W8D9.

## Introduction

Suicide is a significant concern among older adolescents, ranking as the fourth leading cause of death, worldwide (1). Due to heightened risk-taking behaviors and mental health challenges, instances of suicidal thoughts and behaviors (STBs) are more prevalent during adolescence (2, 3). Moreover, suicide prevention, unlike road safety, currently lacks a comprehensive strategic framework to effectively reduce mortality and morbidity. This gap is partly attributed to the insufficient evidence base surrounding potentially successful strategies across diverse settings. Although previous reviews have evaluated youth suicide and self-harm prevention initiatives within specific settings such as schools or clinical environments, there is currently no recent, comprehensive synthesis of the outcomes across these intervention-based strategies (4). Interventions aimed at preventing youth suicide have been deployed across various settings, such as schools, communities, and healthcare systems around the world (5). However, they are tailored to diminish risk factors associated with suicidal behavior or to identify individuals at risk and facilitate access to treatment or assistance (6). The school-based suicide prevention (SSP), therefore, encompasses various preventive strategies. These include universal approaches, aimed to reach the entire school population and selective strategies, that identify and support at-risk groups showing signs of suicidal thoughts or behaviors (STBs) (7).

The journey from childhood to adulthood requires navigating through diverse developmental milestones and experiences, which exerts substantial influence on the physical, emotional, social, and mental wellbeing of adolescents. Throughout the transition phase, from childhood to adulthood, adolescents undergo an increasing number of mental health issues (8). Globally, suicide ranks as the fourth major cause of death for adolescents and young adults aged 10–19 years (9). Prioritizing mental health interventions during this period is, therefore, essential, to combat the high burden of mental disorders prevailing in this age group (10).

Adolescent suicide remains a significant global public health concern, ranking as the second leading cause of death among individuals aged 15–29 (11, 12). In this age group, the family serves as the primary influencer, shaping adolescents’ beliefs, attitudes, and knowledge. Moreover, this period, marked by profound biological, cognitive, and social changes, is crucial for development, as adolescence is viewed as a transition from childhood to adulthood, which prepares them for adult roles (13). Additionally, Erikson (14) highlights another fundamental factor, the development of personal identity during this stage, which typically occurs in the 12–15 years of age. Besides this, mood swings are also common during adolescence, due to hormonal changes (13).

Suicidal behaviors frequently involve self-harming thoughts (suicidal ideation), suicide planning, and suicide attempts. Latter are a strong indicator of the suicidal risk (15). Suicidal ideation ranges from the notion that life is meaningless, to persistent and intrusive thoughts of self-destruction (16). A meta-analysis of global suicide pooled analysis trends (2010–2020) found an overall rate of 3.77 per 100,000 people, with Estonia, New Zealand, and Uzbekistan reporting the highest rates. Suicide was more prevalent among older adolescents and males (17). Only 7% of those experiencing suicidal thoughts made an attempt to take their own life (18). Yet, the occurrence of suicidal thoughts and attempts exceeds the number of actual suicide fatalities (19). Research indicates that for every suicide death, there are about 50–100 suicide attempts among young individuals (20). Both suicide attempts and severe suicidal ideation can lead to significant consequences, such as deep psychological impact, heightened risk of future suicide attempts, and even death (19).

The range of suicide prevention interventions in the review consisted of primary prevention, such as public awareness initiatives; secondary prevention, like gatekeeper training programs; tertiary prevention, including counseling, therapy; and postvention, such as survivor support groups. Expanding the conventional classification, these suicide prevention strategies can be categorized into universal (targeting the general public), selective (aimed at specific groups with elevated lifetime risk (such as, adolescents), and indicated prevention (targeting high-risk) groups with already elevated risk (such as, psychiatric inpatients) (21). Suicide prevention interventions aims to reduce the overall risk and promote mental wellbeing at the population level. Preventive strategies may also encompass direct interventions, particularly during crises, to prevent imminent suicide risk (22). In this review, suicide-specific prevention (SSP) focuses on addressing suicidal thoughts and behaviors (STBs) as primary outcomes, while pursuing broader health, and wellbeing as secondary outcomes.

Given the limited organized knowledge on the implementation of suicide prevention interventions for school-age adolescents, the goal was to identify and chart the empirical literature on the strategies of SPI as well as outcomes for this age group. Previous reviews have explored suicide prevention interventions within specific settings, such as schools (23) or healthcare (24), or focused on particular approaches like clinical or community-based programs. However, a comprehensive analysis of intervention outcomes across multiple settings, along with key implementation barriers and facilitators, is lacking.

This review fills that gap by systematically mapping suicide prevention strategies for school-age adolescents, emphasizing their impact on suicidal behaviors and mental wellbeing. Unlike prior reviews that focus on a single setting, this current study synthesizes cross-sector interventions and highlights contextual factors shaping implementation. By identifying barriers and facilitators, we provide insights to guide policy and practice across diverse economic and healthcare contexts. The findings could guide the development and design of best evidence base effective interventions to reduce suicidal behavior and improve mental wellbeing among youth.

## Methods

This study employed a scoping review methodology to assess suicide prevention programs, identify research gaps, and compare interventions and outcomes across different contexts. It aimed to capture diverse strategies to inform school-based suicide prevention efforts. The review process was guided by Arksey and O′Malley’s five-stage framework, which includes formulating the research question, locating relevant studies, establishing selection criteria, organizing data, and summarizing findings (25). This review is registered in open science framework in 2022 ID:osf-registrations-6w8d9-v1.^1^

The PRISMA SCR guideline was used in developing this scoping review (26). The scoping review provided a structured summary, detailing the background, objectives, methods, results, and conclusions in relation to the review questions. The findings were synthesized, linked to key groups, and discussed in terms of relevance, limitations, and future implications. Scoping reviews draw on a diverse range of evidence and research techniques through a clear, methodical, and reproducible process. The proposed scoping review will follow the standard protocols outlined in the JBI Manual for Evidence Synthesis, including six phases: (1) outlining objectives and research questions, (2) developing inclusion criteria, (3) formulating the search strategy, (4) selecting data sources, (5) extracting information, and (6) analyzing and presenting the results.

## Inclusion criteria

### Participants

Following the WHO adolescent age range, this review will include studies that focus on school-aged adolescents between the ages of 10 and 19 years (27). Eligible studies specifically targeted school age adolescents, without restrictions based on gender, ethnicity, or other sociocultural factors.

### Concept (C)

The primary focus was on the implementation and effectiveness of suicide prevention interventions (SPI) for school aged adolescents. Eligible interventions included educational, supportive, counseling, therapeutic, and psychological strategies, while pharmacological interventions were explicitly excluded. Outcome measures assessed changes in suicidal behaviors (Self injuries, self-harm attitudes, suicidal thoughts, and completed attempts) and overall mental wellbeing. Eligible studies were scholarly, peer-reviewed articles that detailed an intervention study with pre-and-post outcome assessments, focused primarily on suicide prevention interventions targeting any type of suicidal behavior, involved school-aged adolescents (10–19 years), and were published in English between 2015 and February 2025.

### Context (C)

The review included studies from all geographical regions, focusing on interventions implemented within school settings in both high income countries and low- and middle-income countries (LMICs). No restrictions were placed on provider type, delivery methods, duration, or implementation strategies. A systematic approach was employed to examine and compare suicide prevention strategies across diverse contexts, assessing intervention effectiveness through narrative synthesis. Both quasi-experimental studies and randomized controlled trials (RCTs) were included, given their ability to provide robust evidence. Systematic and scoping reviews were also considered to capture broader insights into existing intervention strategies. However, qualitative studies, cross-sectional studies, study protocols, pilot studies, conference proceedings, and non-peer-reviewed articles were excluded to maintain methodological rigor.

#### Identifying the research question

The review sought to address the question,

1. What is the current evidence on suicide prevention interventions for school aged adolescents?
2. How do these interventions impact outcomes suicidal behaviors (attitudes, thoughts, and attempts) and mental wellbeing?
3. What are the key barriers and facilitators in implementing suicide prevention strategies for school-age adolescents across different contexts?

This question was framed by merging a broad research question with a clearly defined scope (25, 28). In this context, the search encompassed any intervention or program that aimed at preventing suicide and/or improving wellbeing among adolescents aged 10–19 years and had its program details published in international peer reviewed journals.

#### Identification of relevant studies

In February 2025, a comprehensive search of five different databases Pub Med, EBSCO, Science Direct, Psych-info and ProQuest. Then, gray literature searches will be conducted through the Open Gray, Gray Matters, and Google Scholar platforms, using the Chrome browser in private browsing mode to lessen geographical bias. Finally, we explored the reference lists of systematic reviews to uncover further relevant studies. Two researchers reviewed the identified articles (YP, RN and MA). The selection process was guided by increasing familiarity with the literature, and by applying the inclusion criteria iteratively. The PRISMA-ScR tool was utilized for transparency in reporting.

### Study selection process and criteria

A comprehensive literature review was conducted to examine the research topic from multiple angles. Data were retrieved using Boolean operators (AND, OR) with a combination of key terms; for example, (“Prevention Program” OR “strategies” OR “intervention” OR “measures”) combined with (“Adolescent” OR “Teenage” OR “school age”), along with terms related to self-injurious behavior and suicide (e.g., “suicide,” “suicidal,” “suicidality,” “Suicidal Behaviors”) and mental health aspects (e.g., “mental wellbeing,” “mental health,” “psychological health”), further paired with (“interventional” OR “Reviews”). A 20-year filter was applied. For Science Direct, the search was modified to “Suicide” AND “Mental Wellbeing” AND (“Prevention Program” OR “intervention”) AND (“Adolescent”) (see Appendix).

A total of 1,571 articles were initially obtained, after applying additional filters: English language, last 20 years, human subjects, adolescents, and full text, Of these, 961 articles were removed from various databases, leaving 610 articles after duplicate removal and screening titles. After applying the inclusion and exclusion criteria and screening abstract, 87 publications were determined to be relevant and included in the review. The 87 articles were then screened full text in detail, which narrowed the pool to 62 articles. Following this, 25 articles were excluded because they did not address the specific research topic, lacked rigorous methodology, or did not align with the required study types, with some being dismissed for insufficient data or low quality.

**Figure 1:**
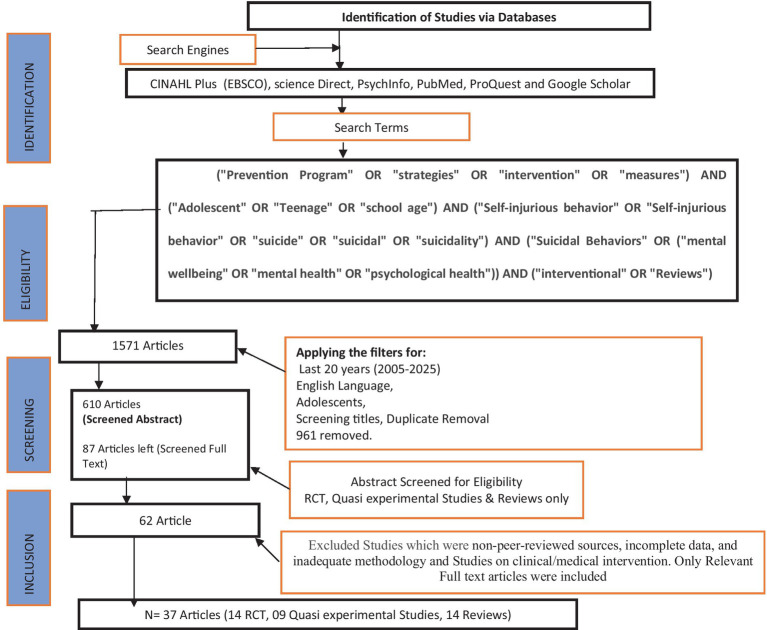
Summary of search strategy process.

Ultimately, 37 articles were fully reviewed to extract meaningful data. These articles included 14 quantitative studies, comprising, 11 randomized controlled trials (RCTs), 3 quasi-experimental studies and 11 reviews (including systematic and meta-analytic studies) that synthesized evidence from multiple primary studies to assess the effectiveness of various interventions. Figure 1 represents a summary of the search strategy process.

#### Charting the data

Data from studies were compiled using a descriptive analytical approach, which is commonly used in scoping reviews. The information was compiled into an Excel spreadsheet, recording key details such as author(s), location, purpose, study design, participants, outcomes, findings. Specific program details were also gathered, including the program name, type of intervention, target audience, duration, frequency, requirements, delivery methods, and expected outcomes.

#### Collating, summarizing, and presenting the findings

In scoping reviews, the stage of gathering, synthesizing, and presenting findings is essential for compiling and interpreting extracted data to provide a broad understanding of the research landscape. This process typically involves both quantitative and qualitative analyses (29). Quantitative analysis includes measuring the distribution of studies based on factors such as publication year, geographic origin, or research methodology, often illustrated through tables or graphs to emphasize key aspects of the review (30, 31). Narrative analysis entails reviewing textual data to identify recurring themes or patterns related to the research question, using coding techniques to categorize these insights. This combined approach ensures that the results are methodically structured and clearly aligned with the review’s objectives, offering meaningful insights into the existing body of research. After generating the codes, the research team refined them further through discussions (32).

### Data analysis

Data was collected from the studies included in the scoping review by two independent reviewers using a data collection template. It includes information on study identifiers, such as the first author, title, year of publication, country of study, and article type. The form also collects data on the type of intervention or prevention, including its preventive components, clinical outcomes, frequency and duration, setting, and target audience. In addition, the methods section captures the aims and objectives of the study, as well as its design. Information on the measures or tools used in the study, along with process and sample characteristics, is also documented. Finally, the results section gathers details on characteristics and outcomes, process outcomes, fidelity or adaptation of the intervention, any contextual changes, facilitators, barriers, potential harms, and recommendations for future research.

Narrative synthesis was employed to summarize the findings by: (1) constructing an initial synthesis of the included studies by organizing data into codes that enhance the researchers’ ability to detect patterns across studies; (2) analyzing relationships within the data while considering various factors that could influence the magnitude or direction of the intervention’s effects across studies; and (3) evaluating the reliability of the data by discussing its strengths and limitations, as well as identifying study-specific factors or implementation barriers that may account for inconsistencies in findings (33).

## Results

The results from the reviewed studies encompass a total of 37 research papers, including 14 randomized controlled trials (RCTs), 14 review including systematic reviews, 9 quasi-experimental studies, and pretest-posttest studies. These studies span multiple regions, providing insights into adolescent mental health and suicide prevention across diverse cultural and socio-economic contexts. In Europe, research has been conducted in the Netherlands, the UK, Germany, Austria, France, Hungary, Ireland, Italy, Romania, Slovenia, Spain, and Sweden. In Asia, studies cover India, China, Lebanon, Jordan, Bengkulu, and broader Upper and Low Middle-Income Countries.

North America is represented by research from the United States, while Australia has contributions from New South Wales and Victoria. Additionally, multinational studies, such as the SEYLE project, have examined interventions across 11 European nations, further enriching the global understanding of effective mental health strategies for adolescents.

A significant gap identified in the literature was the scarcity of research carried out in LICs and LMICs. Majority of the studies included in this review were conducted both in high-income countries (HICs), focusing on adolescents from relatively homogeneous socioeconomic backgrounds. Hence, most of the evidence gathered originates from high-income countries, as depicted in Table 1, potentially limiting its applicability to other regions. Suicide and/or self-harm prevention training varied among the studies, typically spanning either from half to a full day, or sometimes 1–2 days. The modular delivery methods were common, often incorporating didactic training, supplemented by group work, scenario based discussion, and individual or group reflection. Moreover, trainers predominantly possessed backgrounds in psychology or healthcare.

**Table 1:** Focus of suicide prevention programs.

	Suicidal ideation/planning	Suicidal attempt	Suicidal behavior and action	Suicidal death	Self-injury act/risk	Suicidal stigma	Health-seeking intention/behavior	Suicide literacy
Braun et al. (54)	y		y			y	Y	
Grande et al. (12)	y	y			y			
McGillivray et al. (115)	y	y				y	Y	y
Fonseca Pedrero et al. (55)	y	y	y		y			
Singer et al. (37)			y					y
Bradley and Toole (116)	y	y	y					
Grummitt et al. (59)	y							
Babeva et al. (63)		y	y		y			
Wright et al. (64)	y							
Witt et al. (72)		y	y		y			
Davaasambuu et al. (24)	y	y	y					
Jager-Hyman et al. (117)	y	y		y				
Nasution et al. (61)	y							
Roberts et al. (60)	y		y					
Gijzen et al. (56)			y					
Grupp-Phelan et al. (57)	y	y	y					
Kaess et al. (71)	y	y	y					
Lindow et al. (65, 66)	y		y					
Morken et al. (75)	y	y		y	y			
Davis et al. (118)	y		y					
WHO (119)	y		y				Y	y
Davis et al. (118)	y		y				Y	

The training content generally varied across studies; but it typically included defining self-harm and suicide, presenting suicide facts and prevalence statistics, addressing attendees’ attitudes and perceived attitudes of others, and challenging negative attitudes. Training focused on risk factors for self-harm and suicide, including interpersonal relationships, along with risk assessment and mental health screening for healthcare professionals. Across the studies, outcome measures comprehensively captured changes in participants’ attitudes, knowledge, and confidence, while a subset of studies employed validated instruments to assess constructs including depression, anxiety, suicidal attitudes, and ideations. Although statistically significant improvements were observed, the findings were inconsistent, with most studies highlighting enhanced confidence, improved attitudes, and higher trainee satisfaction rather than clear, tangible reductions in suicide or self-harm behaviors.

### Suicide prevention interventions

#### Suicidal literacy and gate keeper trainings

Mental health literacy forms the basis for promoting, preventing, and providing care related to mental health issues, ultimately enhancing mental wellbeing (34). Effective gatekeeper training is ongoing or repeated suicide prevention programs, rather than being carried out through one-time sessions. These trainings typically target the entire adolescent school aged population, and include education on identifying risk factors, warning signs, and appropriate help-seeking behaviors, for themselves and for their peers. In a systematic review of Randomized controlled trials (RCTs) of the Signs of Suicide (SOS) program have shown a decrease in suicide attempts over a 3-month period (35). The Signs of Suicide (SOS) program, a prominent evidence-based initiative, features a comprehensive program focused on enhancing suicide awareness, and identifying key risk factors, such as depression (36). Additionally, a large-scale randomized controlled trial (RCT) of the Youth Aware of Mental Health (YAM) program, which included over 11,110 secondary school students from 10 European countries, demonstrated a 55% reduction in suicide attempts and a 50% decrease in severe suicidal ideation compared to the control groups, 12 months after the intervention (19).

Another program, the Suicide Prevention Initiative (SPI), in Rhode Island, mentions collaborations with clinicians to help the school staff manage student suicide risk; it included three tiers: (1) training school staff in assessing suicide and depression, (2) connecting schools with a clinical care coordinator, (3) conducting follow-up check-ins with families. The coordinated framework enables comprehensive care and monitoring, integrating education and healthcare systems for optimal support (37). Similarly, the Safety Plan Intervention (SPI) is a concise, evidence-based approach that addresses suicide crises with strategic components: reducing lethal means, enhancing problem-solving, coping skills, social support, and emergency contact identification. SPI is a collaborative strategy for managing suicidality and reducing access to lethal means. It includes identifying warning signs, coping strategies, social support, available of emergency contacts and professional resources, and methods to create a safe environment. Moreover, the plan is revisited and revised periodically. On the other hand, Brodsky et al. (38) have proposed Crisis Response Planning (CRP), entailing individuals to list steps for recognizing personal warning signs, using coping strategies, seeking social support, and accessing professional services on a small card. Both these interventions promote prioritizing safety, by discussing suicidal thoughts, impulses, and behaviors upon identifying the warning signs.

#### Suicide prevention intervention reducing suicidal behavior’s

School play a crucial role in providing mental health interventions to adolescents in HICs, where the focus is often upon implementing universal psychosocial programs that aim at enhancing wellbeing and mitigating mental health risks. Universal programs targeting mental health promotion in adolescents can yield positive outcomes. Moreover, evidence indicates that school-based socio-emotional learning (SEL) programs effectively lower depression, anxiety, and suicidal behaviors in adolescents (39). Enhancing adolescents’ awareness of mental health resources and social support, while fostering help-seeking skills, is a critical component of effective suicide prevention in schools, addressing the prevalent reluctance among youth to seek assistance (40–42). Whereas, there is limited research on school-based interventions in LMICs, despite their potential to reach large numbers of adolescents (43). Moreover, in New Delhi, India, a randomized trial evaluated the effectiveness of a concise problem-solving intervention for common adolescent mental health concerns. Conducted in six government schools with 251 participants, the study found that non-specialist counselors, aided by printed materials, achieved notable psychosocial benefits on adolescents (44).

A trial by Bryant et al. (45) with Syrian refugee adolescents in Jordan, revealed that the Early Adolescent Skills for Emotions (EASE) intervention, as compared to the enhanced usual care (EUC), reduced problem internalizing in adolescents, and improved psychological wellbeing and parenting consistency in caregivers. Another trial conducted in Lebanon, on adolescents aged 10 to 14, who were experiencing emotional distress, revealed that the WHO’s EASE intervention, was found to be acceptable and feasible in reducing psychological distress (46).

A multi-center, cluster randomized controlled trial, called the Saving and Empowering Young Lives (SEYLE) was conducted by Feldman et al. (47) in Europe, involved 11,110 adolescents from 168 schools across 10 European Union countries. The schools were randomly allocated to one of three interventions or designated as a control group. The three interventions included: (1) Question, Persuade, and Refer (QPR) gatekeeper training for teachers and school staff; (2) the Youth Aware of Mental Health Program (YAM) aimed at students; and (3) professional screening (ProfScreen) that provided referrals for at-risk students. At the three-month follow-up, no significant differences were observed between the intervention and control groups. However, at the 12-month follow-up, YAM was associated with a significant decrease in the number of suicide attempts (OR 0.45, 95% CI 0.24–0.85; *p* = 0.014) and severe suicidal ideation (OR 0.50, 95% CI 0.27–0.92; *p* = 0.025) compared to the control group. These results underscore the efficacy of YAM in lowering suicide attempts and severe suicidal thoughts among school-based adolescents, highlighting the advantages of this universal suicide prevention program in educational settings (47). Thus, these interventions can be strongly considered as a part of any comprehensive package of public health strategies focused on promoting overall health and preventing diseases. The Mental Health First Aid (MHFA) training in the United-States provided adolescents with skills and enhanced their mental health literacy. Tailored for students, the training focused on recognizing warning signs, initiating conversations, seeking help, involving responsible adults, and responding to crises. Students who received the training demonstrated increased willingness to provide supportive first aid and reduced likelihood of harmful responses, such as ignoring suicidal peers or keeping their thoughts secret (48). These results highlight the efficacy of universal training programs for adolescents, to enhance their ability to identify peers with mental health issues. Moreover, those who received these trainings exhibited improved helpful first aid intents and reduced blaming attitudes toward peers facing mental health challenges.

### Suicide prevention intervention on mental health promotion

Mental wellbeing is essential for optimal mental health, impacting health-seeking behaviors, decision-making, interpersonal communication, and resilience in crisis. Moreover, it plays a vital role in the overall wellbeing of an individual by enhances emotional wellbeing, promoting proactive management of mental illness and fostering a culture of acceptance (49). A pilot study, to assess the impact of the suicide prevention intervention “Kooth,” that focuses on the wellbeing of young individuals by instilling purpose through helping others and by boosting self-help strategies and social confidence in adolescents, was found to be cost-effective preventive mental health support (50). Likewise, a review of 15 studies underscores the benefits of school mental health programs focusing on holistic wellbeing, revealing enhanced mental health outcomes for adolescents when emphasis shifts from prevention to promotion (51). Whereas, school based mental health interventions are mostly school-based life skills and resilience programs, which have shown positive effects on students’ self-esteem, motivation, and self-efficacy (51).

Furthermore, a four phase LEAP (Learn, Explore, Assess Your Options, and Plan) intervention, rooted in the cognitive-behavioral theory, was designed to address perceived burdensomeness among adolescents (52). In the Learn phase, adolescents were taught how thoughts, emotions, and actions influence each other, and that these can be intentionally modified to promote positive mental health. During the Explore phase, adolescents were taught to identify individuals and situations that felt like a burden. While, during the “Assess Options” phase they were involved in challenging distorted cognition through reality-checking, and by scheduling activities that were aimed at reducing perceptions of burdensomeness. In the Plan phase, adolescents were asked to schedule specific activities, and to plan reminders to ensure completion, which was likely to increase the likelihood of completing the planned activities (52).

Yet another study was done by Pannebakker et al. (53), to evaluate the impact of the Skills 4 Life (S4L), Social Emotional Learning program on secondary school students. The intervention focused on mental wellbeing and associated risk factors, such as self-esteem, self-efficacy, and social skills. The initial four lessons of the S4L curriculum aimed to introduce students to its center rules, by enhancing their cognizance of thoughts, feelings, and behavior, providing alternatives for thinking, and correcting illogical thinking. The program was found to be particularly effective for adolescents with lower educational levels, emphasizing the value of implementing Social Emotional Learning programs for this vulnerable group.

Refer to Tables 2, 3 for details and Nature of intervention in empirical and non-empirical record.

**Table 2:** Details of Interventions in empirical records.

Type of intervention	Nature of intervention	Strategies	Details of intervention	Frequency of intervention	Outcomes	Age	Location	Country	References
Selective Prevention (targets at-risk groups)	Personality-targeted Psycho education selective prevention	Face-to-face sessions in secondary school	Psycho-education on personality traits, identification of maladaptive coping behaviors, and cognitive-behavioral training to reshape unhelpful thought patterns	Two sessions, each of 90-min one session per week	Anxiety and Depression (Brief Symptoms Scale)	13 years	School Based	New South Wales	(59)
	Mind and based Intervention	Talking about mental health, identifying risk, communicating, managing emotions, and making changes and reviewing progress	Focused on mental health awareness, risk identification, effective communication, emotional regulation, behavior change, and progress evaluation	8 weekly group sessions and 3 one-to-one sessions	Strengths and Difficulties Questionnaire, Short (WEMWBS)	13-17 years	School and community Based	UK	(60)
	Cognitive Behavioral therapy and peer leadership	NA	NA	NA	Beck Scale for Suicide Ideation	Grade XI	School based	Bengkulu	(61)
Indicated prevention (targets individuals showing early symptoms or high risk)	Motivational Interviewing Text Messages Individualized safety plan	Face-to-face sessions Mobile Technology	Safety planning sessions with teens and their families, supportive text messages to encourage coping and safety, and follow-up calls to help teens stay on track and support parents after discharge	Text messages till 1 month after discharge	Columbia-Suicide Severity Rating Scale, Self-Efficacy	13-17 years	Clinical/ Hospital Based	USA	(62)
	SAFETY intervention	Individual parent and family sessions SAFETY supports by therapist both parents and adolescents	DBT-based, family-focused therapy that follows a two-therapist approach to assist teens and caregivers in managing suicidal behaviors by strengthening support, resilience, and emotional control. It incorporates cognitive-behavioral techniques, customized safety planning, and a structured model (SAFE pyramid) to boost protective factors and ensure ongoing care	12 weeks	Harkavy Asnis Suicide Survey, Center for Epidemiological Studies-Depression Scale, Beck Hopelessness Scale	11–18 years	Hospital Based	USA	(63)
	Telehealth education	Guides (iPad-based audiovisual connections) for enhancing psychological wellbeing	Audiovisual group sessions for HPN patients, guided by a psychologist, covering mental health skills, depression prevention, mindfulness, problem-solving, and transition to adult healthcare. Participants practiced coping strategies using discussion-based learning and interactive health-related apps.	35 sessions (three weekly 1–1.5 h-long group) audiovisual discussion sessions conducted using encrypted.	Weekly audiovisual group sessions	13-30 years	telehealth- home based for HPN patients	USA	(64)
	(STAT-ED) intervention	Motivational interviewing to target family engagement, problem solving, referral assistance.	EUC intervention received a standard mental health evaluation and referral in the emergency department (ED). In contrast, the STAT-ED intervention included motivational interviewing, barrier reduction discussions, brief case management, and follow-up calls to support mental health treatment engagement.	A supportive telephone call within 2 days of discharge, a call the day before the scheduled appointment, and 1 or 2 follow-up calls	Suicidal Ideation Questionnaire, CES Depression Scale	13-17 years	Hospital based	Columbus	(57)
Universal prevention (targets the general population)	Multimodal school-based Intervention	The intervention consists of phase 1 screening, phase 2: gatekeeper training, phase 3: universal and phase 4: indicate prevention. Phase 2: training will be based on the Question, Persuade, and Refer (QPR) gatekeeper training, Phase 3: The third module “universal prevention” focused on “Moving Stories” and games.	A multimodal stepped-prevention program with screening, gatekeeper training, universal prevention using a serious game, and a CBT-based indicated prevention module for high-risk students. It aims to identify, educate, and support adolescents at risk for suicide and depression while integrating school-based mental health strategies.	Consisted of 8 lessons of 60 min each.	Anxiety Disorder Interview Schedule for Children, Depression Stigma Scale	15-29 years	School Based	Dutch	(56)
	Youth Aware of Mental Health (YAM) and Signs of Suicide (SOS) program	School-based classroom sessions and booklet	School-based mental health program delivered in five sessions using role-plays, discussions, and educational materials to enhance awareness, problem-solving, and help-seeking regarding mental health and suicide prevention. It was implemented in secondary schools as part of the LifeSpan trial, ensuring a safe environment for students to engage in meaningful conversations about mental wellbeing.	Five sessions over 3 weeks	General Help-Seeking Questionnaire (GHSQ), LOSS	12–17 years	School based	Australia	(57, 115)
	Saving and Empowering young lives (SEYLE)	Three interventions include gatekeeper training (QPR), awareness training on mental health promotion for adolescents, and screening for at-risk adolescents by health experts Prevention intervention II. Awareness Training of Adolescents regarding suicide: All adolescents are provided with a tailored educational, awareness-raising booklet covering enhancement psychosocial capabilities in schools	Gatekeeper Training (QPR) for teachers and school staff to identify and refer at-risk students, Awareness Training to educate students on mental health, stress management, and crisis response, and Professional Screening to assess and refer students with high-risk behaviors for clinical evaluation. These strategies aim to enhance psychological wellbeing and reduce suicidal risk, compared to a minimal intervention control group with only educational posters	Gatekeeper training consisted of a two-hour interactive lecture followed by a one-hour role-play session.	At baseline and at a 12-month follow-up, all adolescents completed wellbeing, strengths and difficulties, depressive symptoms, and suicidal behavior. Beck Depression Inventory, Global School-Based Pupil Health Survey, SDQ, PSS,	12-15 years	School Based	Austria, Estonia, France, Germany, Hungary, Ireland, Israel, Italy, Romania, Slovenia, Spain, and Sweden	Wasserman et al. (58)
	School-based youth aware of mental health (YAM) program	Roleplay, lecture, an information booklet for students, and six posters.	Five sessions with role-play, interactive lectures, and supporting materials to enhance mental health awareness, self-help strategies, and crisis management. Certified facilitators (non-school personnel) delivered YAM in a safe, open discussion format, focusing on Mental health awareness; self-care guidance, stress and crisis management, depression and suicidal ideation, supporting a struggling friend, and information on mental health resources and seeking help, adapting it linguistically and culturally for the U.S. context	Three role-play sessions Two mental health interactive lectures (50 min each), over 3–5 weeks	General Help Seeking Questionnaire	12–18 years	School Based	USA	(41)

**Table 3:** Nature of interventions in non-empirical records.

Intervention	Meaningful statement and clustering study code	Sub-categories and categories
Cognitive, social approach Community-based framework	Cognitive behavioral intervention (12, 72, 75, 116) Psycho-social intervention (12) Family interventions (72, 75), Public awareness campaign (75, 116)	Individual and community-based Psychosocial suicide prevention programs Cognitive behavioral intervention (12, 72, 75, 116), Psycho-Social intervention (12, 75, 118) Family interventions (72, 75), Public awareness campaign and skill building (75, 116, 118, 119) Remote contact interventions (72) self-management of suicidal ideation and self-harm (75) Safe Media Messaging (118, 119)
Interpersonal theory of suicide (IPTS). Integrated motivational-volitional model (IMV) and the three-step theory (3ST) the fluid vulnerability theory (FVT)	Interpersonal theory of suicide (IPTS) (55) Integrated motivational-volitional model (IMV) (55) Three-step theory (3ST) (55) The fluid vulnerability theory (FVT) (55)	Theory/framework guided suicide prevention programs Interpersonal theory of suicide (IPTS) (55) Integrated motivational-volitional model (IMV) (55) Three-step theory (3ST) (55) The fluid vulnerability theory (FVT) (55)
Safety planning intervention. Brief intervention led by doctoral-level staff Restricting access to lethal means Training health and human service providers	Reducing access to methods commonly used for suicide, such as firearms, pesticides, or medications (119) training healthcare professionals, social workers, and other service providers to recognize the signs of suicidal behavior, assess risk, and provide appropriate care and support to individuals at risk (119)	Safety enhancement suicide prevention program Safety planning intervention (37, 117). Restricting access to harmful means (118, 119) Increased accessibility of youth to suicide prevention Centre (118) Suicide Screening at healthcare centers (118)
Individual CBT-based psychotherapy. Dialectical behavioral therapy. Mentalization therapy. Group-based psychotherapy; Enhanced assessment approaches; Compliance enhancement approaches; Family interventions; Remote contact interventions	Remote contact interventions (72)	
Suicide prevention practices or safety planning strategy Safety planning intervention		
Educational Intervention. Zuni Life Skills Curriculum, based on seven main units: building self-esteem; identifying emotions and stress; increasing communication and problem-solving skills; recognizing and eliminating self-destructive behaviors delivered by Non- Zuni female teachers and two trained Zuni male teachers. Other module, Qungasvik intervention consists of 24 modules delivered in two sessions of 1–3 h, focusing on protective factors within individual, family, and community domains. It includes individual mastery of relationships, family cohesion, expressiveness, and conflict, as well as community support and opportunities. Peer influences are assessed using scales from the American Drug and Alcohol Survey, and the program integrates reflective processes and explores reasons for life as protective elements	School-based prevention programs (75)	School-based education programs for suicide prevention School-based prevention programs (75)

Table 1 offers a comprehensive overview of the focus areas embedded in various suicide prevention programs, categorizing each study based on whether it addressed dimensions such as suicidal ideation/planning, suicidal attempt, suicidal behavior/action, suicidal death, self-injury risk, suicidal stigma, health-seeking intention/behavior, and suicide literacy (with a “y” indicating inclusion). The analysis reveals a predominant emphasis on early intervention, as evidenced by the majority of studies (54, 55) focusing on early-stage indicators like ideation and attempts, suggesting that these programs are designed primarily to detect and intervene before the escalation of suicidal behavior. In contrast, fewer studies address later-stage outcomes such as suicidal behavior/action and suicidal death, likely due to the challenges in measuring these outcomes or a deliberate focus on earlier prevention. Moreover, while some programs incorporate dimensions of self-injury risk and psychosocial factors such as suicidal stigma, health-seeking behavior, and suicide literacy, these elements are not uniformly represented, indicating that although they are recognized as important, they remain underemphasized. This variability in program scope points clear need for integrated approaches in future research that combine clinical indicators with broader psychosocial factors and adopt standardized metrics to better evaluate and compare the effectiveness of suicide prevention strategies.

The scoping review reveals a diverse array of suicide prevention interventions organized by target population and delivery method. Universal prevention efforts target the general population for mental health promotion through multimodal, school-based programs that combine screening, gatekeeper training (e.g., QPR), and interactive educational modules (56), as well as specific programs like YAM, SOS, and SEYLE, which incorporate classroom sessions, role-plays, lectures, and digital videos to promote mental health awareness and behavior change (41, 42, 57, 123). Selective prevention strategies, aimed at at-risk groups, include personality-targeted psychoeducation delivered through face-to-face sessions in schools (59) and mind-based interventions with group and individual sessions (60), as well as cognitive behavioral therapy with peer leadership (61). Indicated prevention interventions focus on individuals exhibiting early symptoms in past or at high risk, utilizing approaches such as motivational interviewing paired with text messages and individualized safety plans (62), family-focused SAFETY interventions (63), telehealth education via audiovisual connections (64), and cognitive behavioral therapy combined with skills training (24). Additionally, the STAT-ED intervention integrates motivational interviewing, family engagement, and structured follow-up calls (61). This spectrum of interventions, varying in strategy and frequency; from weekly sessions to follow-up messaging, underscores the multifaceted and context-specific nature of suicide prevention efforts.

Table 3 categorizes a wide range of suicide prevention interventions in non-empirical records, primarily emphasize preventive, educational, and systemic approaches, underscoring the importance of mental health literacy, resilience-building, and community engagement. A recurring theme is the universal application of interventions, advocating for school-wide implementation rather than targeting at-risk individuals alone. The literature supports gatekeeper training for educators, structured socio-emotional learning (SEL) programs, and multi-tiered interventions, such as the SEYLE model, which integrates awareness, screening, and referral mechanisms. Additionally, emphasis is placed on cross-sector collaboration, highlighting the role of teachers, caregivers, and healthcare professionals in a holistic, school-based mental health framework.

### Barriers and facilitators of suicide prevention programs

There are several barriers in the implementation of suicide prevention programs for adolescents. These include individual, organizational, socio-cultural, and ethical barriers. Individual barriers can be sleeping problems, chronic illness, engagement in self-harming behaviors, and developmental disabilities. Organizational barriers encompass limited availability of qualified personnel, brief follow-up periods, and lack of specialized training for teachers and counselors (65). Socio-cultural barriers include inadequate parental involvement, stigma surrounding mental health issues, and differing perspectives between parents and adolescents. Ethical barriers include concerns about privacy, confidentiality, and obtaining parental consent for young adolescents (Tables 4, 5). These barriers can hinder the effectiveness and accessibility of suicide intervention efforts (66).

**Table 4:** Barriers and facilitators of suicide prevention programs.

Barriers Personal barriers Adolescents with chronic illness Low engagement in intervention Organizational barrier Non-specialized personnel for provision of suicide intervention Unsustainable suicide intervention Scarcity of resources Socio-cultural barriers Culturally incompetent suicide intervention Fear of social isolation Variations in perspectives
Facilitators Personal facilitators Tailored suicide intervention Individualized approach Organizational/institutional facilitators Sustainability Inclusion of digital health Capacity building Multidisciplinary approach Socio-cultural facilitators Family involvement Community and school based programs

**Table 5:** Data extraction table.

Data extraction sheet							
	References	Country	Purpose	Types of Study	Population	Outcome	Findings
1	Pannebakker et al. (53)	Netherland	To evaluates the impact of the Skills 4 life program on mental health	Cluster randomized controlled	66 classes of 38 schools	Rosenberg Self-Esteem, Strengths and difficulties questionnaire	The Skills 4 Life curriculum demonstrates effectiveness in enhancing mental health and self-efficacy among adolescents, particularly those with lower educational levels
2	Michelson et al. (44)	New Delhi India	To examine the effectiveness of a brief problem-solving intervention for adolescent mental health issues delivered by non-specialist school counselors	Randomized trial conducted	251 participants from six government-run schools	Strengths and difficulties questionnaire	Intervention by school counselors, coupled with printed booklets and delivered by lay counselors, demonstrated a modest impact on psycho-social outcomes among adolescents
3	Stevens et al. (50)	UK	A pilot evaluation to assess its impact of “Kooth” on the wellbeing of young individuals	Pilot Randomized Controlled Trial	302 young adults	Psychological Distress (YP-CORE), Suicidal ideation SIDAS, strengths and difficulties questionnaire, HOPE, KIDSCREEN 10	“Kooth” offers cost-effective preventive mental health support, instilling purpose through helping others, and boosting self-help strategies and social confidence in youth
4	Bryant et al. (45)	Jorden	To evaluate the effectiveness of the Early Adolescent Skills for Emotions (EASE) intervention among Syrian refugee adolescent	Randomized single-blind, parallel, controlled trial	Adolescent aged 10–14 years, 1,842 screened for eligibility, 471 agreed to participate and randomly assigned to receive either EASE or enhanced usual care (EUC)	Warwick Edinburgh Mental Wellbeing Scale (WEMWBS) Psychological Sense of School Membership (PSSM) Scale Kessler Distress Scale (K6) Alabama Parenting Questionnaire (APQ)	EASE effectively reduced internalizing problems (estimated mean difference 0.69 and effect size, 0.38) among young refugee adolescents and improved psychological wellbeing and parenting consistency in caregivers
5	Shahtahmasebi (13)		To explore the effective suicide reduction intervention	Review			The findings emphasize community engagement and public discourse. Strategies include fostering a sense of community concern and promoting open communication with children and neighbors to address various risk factors and challenge the notion of suicide as a viable option
6	Brown et al. (46)	Lebanon	A feasibility trial assessed the effectiveness of the WHO’s Early Adolescent Skills for Emotions (EASE) intervention for adolescents aged 10 to 14 with emotional distress.	RCT, Participants, identified through the Child Psycho-social Distress Screener, were randomly assigned to receive either EASE or enhanced treatment as usual (ETAU)	EASE comprised seven group sessions for adolescents and three for caregivers, while ETAU involved a single psycho-education home visit	Pediatric symptom checklist	Outcomes were evaluated at baseline, endline (8 weeks post-randomization), and three-month follow-up (20 weeks post-randomization). The EASE intervention and study procedures were generally well-received and safe for adolescents and caregivers in North Lebanon. On Qualitative evaluation, Positive feedback regarding the intervention highlighted its impact on adolescent behavior, emotions, and family relationships. Participants noted direct benefits from EASE strategies and increased support from caregivers
7	Grosselli et al. (40)	Germany	To assess the effectiveness, acceptance, and safety of the HEYLiFE suicide prevention program in enhancing help-seeking behaviors and reducing stigma toward peers with suicidal thoughts	Randomized-controlled trial with a wait list-control group	165 German secondary Schools	Signs-of-Suicide-knowledge-questionnaire, Signs-of-Suicide-attitude-questionnaire, General Help-Seeking Questionnaire (GHSQ), PHQ-9, Strength and Difficulties Questionnaire	HEYLiFE program assessment highlights short-term effectiveness in improving knowledge, attitudes, and reducing suicidality risk factors and social distance, demonstrating its potential to prevent suicidal thoughts and behaviors among adolescents
8.	Das et al. (51)		A systematic review was conducted to explore mental health interventions targeting adolescents	Systematic review	The review identified various intervention modalities, including 12 school-based, 6 community-based, 8 digital platforms, and 12 individual−/family-based intervention	38 studies	Findings from school-based suicide prevention programs indicate that classroom-based didactic and experiential approaches enhance short-term knowledge of suicide (SMD: 1.51) and knowledge of suicide prevention (SMD:0 0.72), though no discernible impact on suicide-related attitudes or behaviors was observed
9	Perry et al. (70)		To identify online and mobile psychosocial suicide prevention interventions for young individuals and assess their effectiveness	Systematic review	4 databases were electronically searched for peer-reviewed journal articles published from January 2000 to May 2015		The moderate effect sizes observed for suicidal ideation (*η_p_*^2^ = 0.66) and clinician-rated depression (*η_p_*^2^ = 0.60) in young people following online suicide prevention interventions. These findings justify the need for further research in this area. Current evidence on online and mobile interventions for youth suicide prevention is insufficient, necessitating more high-quality empirical research to assess their effectiveness
10	Torok et al. (74)		To examine the effectiveness of suicide prevention training for parents and teachers, aimed at strengthening their capacity to recognize warning signs and respond appropriately to adolescents at risk	Systematic review	13 studies		These programs generally enhance suicide literacy, evidence for behavioral changes is limited. To improve outcomes, expanding the reach of gatekeeper training through digital platforms and targeting strategies to engage parents, who are crucial sources of support for vulnerable youth, is recommended
11	Asarnow and Mehlum (73)		To assesses scientific evidence on steps in the care pathway for identifying and treating youths at risk of suicide/self-harm, including screening, treatment, and community-level prevention strategies	Systematic review			The evidence suggests that interventions addressing the psychosocial environment and empowering trusted adults to support youths, along with meeting youths’ psychological needs, offer significant benefits in reducing suicide risk
12	Wasserman et al. (120)	Europe	To assess the effectiveness of school-based preventive interventions for suicidal behaviors.	Multicenter, cluster-randomized controlled trial	11,110 adolescent pupils aged 15 from 168 schools across 10 European Union countries. Schools were randomly assigned to one of three interventions or a control group	Paykel Hierarchical Suicidal Ladder, Strengths and Difficulties Questionnaire (SDQ)	At the 12-month follow-up, YAM significantly reduced suicide attempts (OR 0.45, *p* = 0.014) and severe suicidal ideation (OR 0.50, *p* = 0.025) compared to the control
13	Singer et al. (36)		To explore effective approaches and empirical evidence supporting school-based suicide prevention efforts while proposing a framework for their integration into multi-tiered systems of support (MTSS)	Systematic review			The school personnel collaborate with mental health professionals to implement best practices. These professionals can provide ongoing program evaluation, while researchers can address current research limitations through outcome studies
14	Brodsky et al. (38)		This review highlights key areas essential for translating suicide prevention research into clinical practice. These include risk assessment, means restriction, evidence-based treatments, population screening with a continuum of care, monitoring, and follow-up	Systematic review			The Assess, Intervene, and Monitor for Suicide Prevention (AIM-SP) model as a practical guide for implementing ZS practices in clinical settings. This model outlines 10 steps for clinical management, aiming to integrate seamlessly into standard practice to enhance risk assessment, provide brief interventions for safety and coping skills, and ensure ongoing monitoring during care transitions and high-risk periods
15	Marraccini and Brier (121)		To explores the relationship between school connectedness and suicidal thoughts and behaviors among adolescents, recognizing schools as crucial settings for intervention	Systematic-review and meta-analytic study			Findings from 16 samples suggest that higher school connectedness is associated with reduced reports of suicidal ideation and attempts across general, high-risk, and sexual minority youth populations
16	Lindow et al. (65, 66)	Montana and Texas	To assess the potential of the Youth Aware of Mental Health (YAM) program to reduce suicidal ideation, attempts, and suicide	Uncontrolled, pretest/post-test design	11 schools, involving 1,878 students, with a subset of 436 students completing surveys before and 3 months after the intervention	General Help Seeking Questionnaire	The results showed significant improvements in help-seeking behaviors, mental health literacy, and reduced mental health-related stigma post-intervention of the YAM program
17	Hill and Pettit (52)	Florida	To explores the efficacy of the LEAP intervention, a web-based selective preventive suicide intervention targeting perceived burdensomeness in adolescents	Pilot randomized controlled trial	80 adolescents	Interpersonal Needs Questionnaire	These findings support the potential of the LEAP intervention in reducing perceived burdensomeness among adolescents, indicating the feasibility of modifying this construct through a psychosocial intervention. The LEAP intervention, rooted in the interpersonal-psychological theory of suicide (IPTS), aims to mitigate suicidal ideation in adolescents by addressing perceived burdensomeness, which is considered a modifiable factor through cognitive-behavioral principles delivered online
18	Hart et al. (69)		This study aimed to evaluate a novel mental health literacy program, Teen Mental Health First Aid, in Australian high schools	Cluster randomized crossover trial	1,605 students aged 15–17, who received either Teen Mental Health First Aid or a control physical first aid course.	Mental Health Knowledge Assessment, Stigmatizing Attitudes Questionnaire: Confidence and Intentions to Help Scale	Results showed students receiving Teen Mental Health First Aid demonstrated improved recognition of suicidality and intentions to assist a peer at risk of suicide compared to those receiving physical first aid. Although some participants reported transient distress after training, there was no evidence of lasting psychological harm. These findings suggest the program’s effectiveness in enhancing peer support for adolescents at risk of suicide, with minimal adverse effects
19	Braun et al. (54)	Austria	To test a suicide prevention video’s intervention effects on 14–19-year-olds.	Quasi experimental study	299 participants	Reasons for Living Inventory for Adolescents, General Help-seeking Questionnaire, Cognitions Concerning Suicide Scale, Stigma of Suicide Scale, Affective State Scale	The intervention immediately reduced suicidal ideation, with sustained help-seeking intentions at T3. This suggests the effectiveness of narratives featuring peers’ personal stories
20	Nasution et al. (61)	Bengkulu	To assess the effects of cognitive behavioral therapy (CBT) and peer leadership on suicidal ideation among adolescents in senior high school.	Quasi-experimental Study	86 participants	Beck Scale for Suicide Ideation	The training received by mental health nurses, cognitive behavioral therapy, and peer leadership are recommended for prevention of adolescent suicidal ideation in adolescent (*p* < 0.05)
21	Grupp-Phelan et al. (57)	Columbus	To examined a motivational interviewing–based intervention—STAT-ED (motivational interviewing, family engagement, problem-solving, referral assistance, limited case management) verses Enhanced Usual Care (EUC; brief mental health consultation, referral) increases linkage of adolescents to outpatient mental health services and reduces depression symptoms and suicidal ideation	Randomized control Trial	166 participants	Suicide Ask Screening questionnaire	The STAT-ED intervention, incorporating motivational interviewing and family-focused support, outperformed enhanced usual care (EUC) in boosting mental health treatment initiation and attendance among suicidal teens at 6-month follow-up. The intervention is not effective in suicidal ideation or depression over the 6-month period but effective in initiating long term mental health treatment
22	Kaess et al. (75) and Wasserman et al. (2010)	11 countries including Austria, Estonia, Germany, France, Hungary, Ireland, Israel, Italy, Romania, Slovenia, and Spain implemented the SEYLE study, with Sweden	To assess the impact of a two-stage school-based screening on service use and suicidality among adolescents from 11 European countries after 1 year	RCT with 2 groups	12,395 participants in the (SEYLE) study, 516	Beck’s Depression Inventory, PSS, WHO Wellbeing Scale, Strengths and difficulties Questionnaire	Screening completion was associated with higher service use (OR 2.695, se 1.017, *p* ≤ 0.01) and lower suicidality at follow-up (OR 0.505, se 0.114, *p* ≤ 0.01) after controlling for potential confounders
23	Morken et al. (75)		To evaluate the effects of interventions preventing self-harm and suicide in children and adolescents	Systematic Review	8 reviews		School-based interventions have been shown to prevent suicidal ideation and attempts in the short term, and potentially reduce the incidence of suicide attempts in the long term
24	Witt et al. (72)		To assess the effects of psychosocial interventions, pharmacological agents, or natural products compared to different types of care for children and adolescents engaging in self-harm (SH)	Review	17 trials with 2,280 participants		Dialectical behavior therapy adapted for adolescents (DBT-A) showed a lower rate of SH repetition compared to treatment-as-usual (TAU), routine psychiatric care, or alternative psychotherapy. Individual cognitive behavioral therapy (CBT)-based psychotherapy did not show a significant difference from TAU. Further evaluation of DBT-A and individual CBT-based psychotherapy is warranted
25	Kong and Kim (90)		To determine the benefits and barriers of school based suicide prevention intervention	Review	18 studies		SPI is effective in lowering suicide risk linked to depression, hostility, and suicidal thoughts while fostering self-worth, social bonds, and gratitude for life. However, obstacles such as insufficient treatment length, absence of follow-up, parental bias, and underdeveloped referral networks impede implementation.
26	Grummitt et al. (59)	NSW and Victoria	To determine the efficacy of selective, personality-targeted prevention in early secondary school	Cluster randomized controlled trial	1,636 students tracked over 3 years	Brief Symptom Inventory	Those receiving preventure demonstrated a significant annual reduction in suicidal thoughts (adjusted OR = 0.80; 95% CI, 0.66–0.97) compared to controls
27	Roberts et al. (60)	England	To assess the efficacy of mind body intervention on Self harm behavior	Quasi experimental study	299 students across 3 places 8 × group session and 3 × individual session 6–8 Student per group	Short Warwick Edinburgh Mental Wellbeing Scale and Strengths and difficulties Questionnaire and Self Harm Risk Assessment	The MAB intervention improved mental wellbeing in 72.8% of students, with notable reductions in emotional (48.5%), conduct (30.5%), and peer issues (32.9%). Additionally, 67% reported fewer self-harm thoughts and 63% fewer self-harm behaviors, highlighting its effectiveness
28	Nasution et al. (61)		To assess the effects of cognitive behavioral therapy and peer leadership on suicidal ideation	Quasi-experimental pre-post-test design	86 participants of high school using purposive sampling	Beck Scale for Suicide Ideation	Suicidal ideation declined from a minimal level to none (*p* < 0.05) after intervention delivered by nurses
29	Czyz et al. (62)		To assess the effectiveness of daily non suicidal self-injury survey on anti-suicide role	Pre- post-test study design	Daily surveys of 80 participants for 4 weeks then follow-up at 1 month and 6 month	Columbia-Suicide Severity Rating Scale	NSSI risk increased on days when ideation was more enduring (OR = 1.99, *p* < 0.001) or intense (OR = 1.66, *p* < 0.001) than usual. Those who attempted suicide within a month post-discharge had higher daily NSSI levels (Hedge’s *g* = 1.26, *p* < 0.001)
30	Babeva et al. (63)		To examine the predictors of treatment response to cognitive behavioral family intervention	Pre- post-test study design	50 adolescents with recent suicide attempts or self-harm	Harkavy Asnis Suicide Survey Center for Epidemiological Studies-Depression Scale and Beck hopelessness Scale	Results showed significant reductions in suicidality, depression, hopelessness, social difficulties, and parental depression with moderate to high effect size
31	Wright et al. (64)	US	To assess the effectiveness of mental health intervention on suicidal ideation and depression using telehealth	Pre- post-test design	80 participants 1.5 h Intervention 3 week	Beck Depression Inventory	Among 25% (*n* = 10) exhibited depressive symptoms or suicidal ideation in pretest, but all showed normalized depression scores post-test, with no suicidal ideation over a year
32	Davaasambuu et al. (24)	LMICs	Evidence Based intervention in reducing suicidal behaviors and depression across 14 LMICs	Review of 22 RCTs	242 combination of CBT, memory based, play, exercise and interpersonal therapy	SSI, BDI, HAM-D	Not any Standalone intervention on reducing suicidal behaviors. New interventions should be carefully designed and assessed to ensure they are culturally appropriate and suited to specific locations
33	Gijzen et al. (68)		To assess interventions targeting known risk factors of suicide-related thoughts and behaviors	Systematic Review and meta-analysis	11 studies with 23,230		School-based suicidal prevention programs have small but significant post-test effects for suicidal ideation (*g* = 0.15) and behaviors (*g* = 0.30). Further research is needed to assess school-based interventions addressing STB risk factors.
34	Kaess et al. (71)	Germany	To assess the efficacy of brief CBT in reducing non suicidal self-injury	RCT 8 motivational sessions	74 participants (ages 12–17) who had self-injured at least five times in the past 6 month	Beck Depression Inventory	CDP is as effective as but more time-efficient than standard treatment (*p* = 0.021)
35	Lindow et al. (41, 42)	US	To assess the efficacy of Youth Aware of Mental Health (YAM)	Pre/post-test design 5 sessions of 50 weeks	11 schools, with 436 Participants	General Help Seeking Questionnaire and Mental Health Stigma Scale	YAM intervention markedly enhanced help-seeking actions, boosted mental health awareness, and diminished stigma at the 3-month follow-up. However, it did not influence the intention to seek help
36	Yang et al. (122)	China	To assess the effectiveness of attention based therapy on depression and suicidal ideation	RCT	45 participants in 8 session	Hamilton depression Scale	Adolescents in the intervention group exhibited significantly lower depression scores compared to control group, *F*(1, 43) = 5.21, *p* < 0.03, Ŋ = 0.11
37	Doty et al. (107)	LMICs	To summarize the evidence of suicide prevention intervention in LMICs	Systematic review	44 studies		Most studies examined universal, selective, and indicated interventions focusing on lethal means or mental health, primarily in (Asia) LMICs. Outcome assessments varied widely across studies. High suicide rates underscore the need for large-scale, youth-focused research to identify effective prevention strategies

Nonetheless, various personal, organizational/institutional, socio-cultural, and ethical, facilitators can help overcome the challenges mentioned above. These include implementing school-based suicide awareness programs, early identification and management of high-risk groups, fostering socio-emotional life skills, and involving families in prevention plans. Additionally, the use of validated screening tools, collaboration among multidisciplinary teams, and the provision of long-term post-discharge support can enhance the sustainability and effectiveness of suicide prevention efforts (67). Furthermore, contextually relevant interventions, digital health tools, and individualized safety plans can address specific needs and promote engagement and ongoing support for at-risk adolescents.

### Thematic analysis

A diverse array of interventions targeting adolescent mental health and suicide prevention has been evaluated using multiple research designs. The studies encompass school-based programs, digital and online approaches, therapeutic/psychosocial treatments, family and community-based initiatives, as well as screening and comprehensive review efforts. Outcomes were measured using standardized instruments such as the Strengths and Difficulties Questionnaire, Beck scales, Hamilton Depression Scale, and various mental health literacy and help-seeking questionnaires.

#### Studies on school-based interventions

School settings emerged as a primary venue for suicide prevention, with numerous studies evaluating structured programs targeting adolescents. The Youth Aware of Mental Health (YAM) program demonstrated effectiveness in reducing suicidal ideation and attempts through psychoeducation, interactive discussions, and role-playing exercises. Other school-based interventions, such as Kooth (50) and Skills 4 Life (53), showed improvements in mental health literacy, self-efficacy, and social connectedness. Similarly, the Early Adolescent Skills for Emotions (EASE) intervention in refugee populations (46) was effective in reducing internalizing symptoms and improving family relationships. A meta-analyses confirmed the small but significant impact of school-based interventions on reducing suicidal ideation and behaviors. However, limitations include challenges in long-term impact assessment and integration into school curricula (51, 68). The Teen Mental Health First Aid program successfully trained students to recognize suicidality and support peers, reinforcing the role of laypersons in crisis intervention (69).

#### Studies on digital and online interventions

The rise of digital platforms has facilitated accessible, scalable interventions for adolescent mental health. Web-based interventions, such as the LEAP program effectively targeted perceived burdensomeness, a key risk factor for suicidal ideation (52). Online and mobile psychosocial interventions showed moderate effects on reducing suicidal ideation and depression, though more rigorous empirical studies are needed to assess their long-term efficacy (70). Telehealth-based interventions demonstrated that telehealth programs significantly reduced depressive symptoms and suicidal ideation, with sustained improvements over a year (64). Similarly The HEYLiFE program improved help-seeking behaviors and reduced stigma, highlighting the potential for digital tools in suicide prevention by (40).

#### Studies on psychosocial and therapeutic intervention

Multiple studies underscored the efficacy of Cognitive Behavioral Therapy (CBT) and its adaptations in suicide prevention. Brief CBT interventions successfully reduced non-suicidal self-injury and suicidal ideation (71). Similarly, Dialectical Behavior Therapy (DBT-A) also demonstrated effectiveness in reducing self-harm behaviors (72). Motivational interviewing-based interventions enhanced mental health treatment initiation but had limited effects on suicidal ideation (57). Similarly, mind–body interventions improved mental wellbeing and reduced self-harm behaviors (60). These findings suggest that structured, evidence-based therapies can significantly impact adolescent suicidality, though program accessibility remains a concern.

#### Family and community-based interventions

Interventions that incorporate family and community dimensions emphasized the importance of community engagement and open discourse in suicide reduction, advocating for strategies that foster a supportive social environment (13). Similarly, the AIM-SP model, a comprehensive framework designed to translate suicide prevention research into clinical practice by integrating risk assessment, brief interventions, and ongoing monitoring (38). In a related vein, (63) utilized a cognitive behavioral family intervention with adolescents who had recently attempted suicide or engaged in self-harm, achieving significant reductions in depression, hopelessness, and suicidal behavior, along with improved parental outcomes. Furthermore, Grupp-Phelan compared a motivational interviewing–based intervention (STAT-ED) to enhanced usual care in a randomized controlled trial, finding that while STAT-ED improved the initiation and continuation of mental health treatment, it did not significantly impact suicidal ideation or depressive symptoms in the short term (57). Furthermore, systematic reviews highlighted the protective role of school connectedness and supportive adult relationships in mitigating suicide risk (73). Programs emphasizing parent and teacher training improved suicide literacy, though evidence for behavioral change remained limited (74).

#### Studies focusing on screening

Screening initiatives and broader systematic reviews have also provided valuable insights. The SEYLE study, implemented a two-stage school-based screening across 11 European countries and found that such screening increased service utilization and lowered suicidality among adolescents (71). Similarly, screening interventions aimed at preventing self-harm and suicide, concluding that school-based approaches can effectively prevent short-term suicidal ideation and attempts (75). Meanwhile, some studies across multiple low- and middle-income countries (LMICs), underscoring the necessity for culturally tailored interventions, as no single strategy was found to be universally effective (24). Furthermore, Czyz et al. (62) highlighted the value of daily self-report surveys in monitoring non-suicidal self-injury, finding that increases in the intensity and duration of suicidal ideation were linked with heightened self-injury risk, emphasizing the need for continuous monitoring to inform timely interventions.

## Discussion

This scoping review provides a comprehensive synthesis of suicide prevention interventions aimed at improving the mental wellbeing of school-going adolescents. The findings highlight the effectiveness of various approaches, including school-based programs, digital interventions, and community-driven strategies. However, suicide prevention remains a multifaceted challenge due to difficulties in achieving long-term impact, reducing stigma, and ensuring the sustainability of interventions (74).

### Barriers in suicide prevention interventions in LMICs

Research consistently shows that school-based interventions can enhance help-seeking behaviors and reduce suicidal ideation among adolescents (76). However, their implementation faces significant barriers, largely due to persistent misconceptions such as the belief that discussing suicide may increase suicidal thoughts (77). Contrary to this concern, evidence suggests that structured screening programs do not heighten distress or suicidal behavior. However, screening for suicidal thoughts and behaviors does not have harmful effects or increase distress (56, 68). Suicide literacy involves understanding its four components; warning signals, causes, risk factors/treatments, and preventive approaches. Ludwig et al.’s study among German adults revealed average literacy with low stigma and minimal normalization (78), while research among Arab youth noted high stigma, limited literacy, and negative views on seeking mental health support (79). Similarly, a cross-sectional study involving 616 participants in Bangladesh revealed that increased understanding of suicide was associated with reduced stigmatizing attitudes (80). Hence, future studies should place greater emphasis on exploring the potential benefits of suicide prevention interventions to increase suicidal literacy and enhancing wellbeing for young populations in developing countries.

A major research gap exists in the underrepresentation of studies from low- and middle-income countries (LMICs), which make up 73% of the global population (81) Despite accounting for 75% of suicides, LMICs receive limited attention in suicide prevention research. Around 90% of suicide cases involve a psychiatric condition, with untreated severe depression being the most common at the time of death (82). Given that two-thirds of countries fall into the LMIC category, there is an urgent need for targeted research and culturally relevant interventions to address depression and suicide in these regions (24).

### Addressing stigma and cultural barriers

Stigma remains a significant barrier to effective suicide prevention. Although increasing public awareness and psychological health literacy has helped diminish the stigma around mental illness, enhancing the availability of mental health services remains essential to effectively support adolescents and young adults (43). Moreover, taboos surrounding suicidal thoughts often instill fear of societal judgment and discrimination among those having such thoughts, which further discourage seeking essential professional intervention (83). These taboos and stigmas influence attitudes and behaviors toward seeking assistance especially in LMICs (41, 42). The stigma surrounding suicide has led to a hidden crisis, preventing many individuals from seeking support for the psychological distress that often precedes suicidal behavior (84).

Research among Arab youth and South Asian communities underscores deeply ingrained stigma and limited suicide literacy, which discourage help-seeking behaviors (85). Suicide prevention efforts must incorporate educational programs to enhance awareness, challenge misconceptions, and foster open discussions about mental health (76). Additionally, culturally tailored interventions such as role-playing exercises, peer-led discussions, and gatekeeper training for educators have shown promise in indigenous and marginalized communities, reinforcing the importance of context-sensitive approaches (86).

### Role of parents, educators, and community engagement

Parents, educators, and communities play a crucial role in suicide prevention. Studies indicate that equipping parents and teachers with knowledge on recognizing warning signs and fostering open communication can significantly reduce suicide risk among adolescents (74). Furthermore, school-based gatekeeper programs have demonstrated effectiveness in improving early detection and intervention (87). Despite their proven benefits, sustaining and systematizing these programs remains a challenge. Strengthening school-based support systems through collaboration with mental health professionals can enhance intervention effectiveness, particularly in resource-constrained settings. Educating parents and teachers on effectively responding to, and supporting, youth experiencing suicidal thoughts or self-harm can play a pivotal role in fostering open communication and reducing suicide risk. As Adolescents are greatly impacted by societal factors, with isolation being a major predictor of self-harm tendencies especially in LMIC’s (88). Nurturing family bonds and stable parental wellbeing lower suicide risk, whereas parental mental disorders and self-inflicted death heighten susceptibility (89).

To alleviate parental apprehensions about revealing suicide risks, school-based SPI should maintain strict confidentiality and participation protocols while ensuring both parents and adolescents are well-informed. Strengthening gatekeeper programs for educators is crucial, as they play a significant role in detecting both risk and protective factors related to adolescent suicide (90). Cooperation among government agencies, communities, religious leaders, schools, and parents is vital for setting long-term objectives and effective strategies for adolescent suicide prevention and intervention. Furthermore, research on school-based SPI must be more systematic and comprehensive.

### Culturally sensitive and contextual relevant program

Various approaches like cultural resource personnel, teacher capacity-building, and culturally tailored role-playing for problem-solving with peer feedback. A study supported this point that culturally adapted suicide prevention programs in schools and communities are particularly vital for indigenous youth, given the limited availability of primary healthcare in indigenous areas (11). Although culturally tailored and community-based psychosocial programs, along with engagement with skilled mental health professionals, could greatly benefit the most at-risk young individuals. However, implementing such strategies may be challenging in low- and middle-income countries (LMICs) due to limited resources and restricted access to qualified healthcare providers and services, suicide prevention in these regions must remain a top priority (91).

Successful cross-cultural adaptation in suicide prevention requires aligning initiatives with local principles, customs, and needs. Nevertheless, low and middle-income nations encounter difficulties in prioritizing suicide deterrence due to scarce assets (92). Merely 10% possess a national suicide prevention framework, and the availability of mental health personnel is considerably lower than in developed nations (91). Given these limitations and the fluctuation of suicide occurrences, regionally suitable, culturally sensitive, and economical solutions are crucial (92).

A study of 134,228 youths aged 14 in LMICs revealed that harassment exposure tripled the likelihood of suicide attempts in 47 out of 48 nations. This connection appeared dose-responsive, with a 12-month attempt rate of 5.9% for non-victims, surging fivefold to 32.7% for those harassed 20–30 days monthly (93). The punishment of suicide attempts in 35 LMICs, with consequences like fines or jail time, is linked to higher suicide rates, especially among women in less-developed countries. Fear of punishment stops people from seeking help, delays medical care, and may lead to more deadly suicide methods and inaccurate reporting (94).

In Pakistan, implementing universal suicide risk screening for adolescents in schools could help early detection, and 24/7 rapid and prompt access services but challenges remain due to stigma, lack of mental health policies, and limited healthcare resources (95). Suicide rates differ significantly between countries, indicating that sociocultural factors may play a role in suicidal behavior. Goldston et al. (96) emphasize the need to consider cultural differences in suicide triggers, responses, help-seeking behaviors, and risk/protective factors. Thus, research should focus on interventions tailored to diverse cultural environments, ensuring that programs are culturally relevant and thoroughly assessed. The absence of culturally sensitive prevention programs in educational settings represents a major limitation, potentially leading to both economic and human costs, highlighting the importance of addressing this gap to develop more effective and inclusive suicide prevention approaches (97).

Gatekeeper training utilizes peer-to-peer support, equipping non-mental health professionals to intervene during suicidal crises, making it a practical approach in resource-limited settings with a shortage of mental health specialists. It aligns with task-shifting strategies, underscoring the necessity for more randomized controlled trials (RCTs) in LMICs (33). Likewise, several universal interventions focus on enhancing help-seeking behaviors and disseminating information about available services, but they assume the presence of accessible and affordable mental healthcare, which may be lacking in certain regions (87).

### Culturally and socioeconomically tailored mental health interventions

Mental health interventions must be tailored to reflect cultural contexts, societal conditions, and specific perceptions of depression and suicidal behaviors. Numerous low- and middle-income countries allocate minimal resources to mental health care, with India investing only $0.22 per capita and Pakistan designating merely 0.4% of its healthcare budget to these services (98). Future research should prioritize examining the potential impact of a country’s economic standing on mental health prevention and intervention outcomes, particularly for adolescents experiencing depression and/or suicidal tendencies (24). Integrating mental health into primary care has several benefits. First, it helps provide more complete, patient-centered care by addressing both physical and mental health needs together. Second, it can reduce stigma and encourage more people and their families to seek mental health support. Additionally, combining therapy with skills like communication, problem-solving, and social development may be more effective than therapy alone (24). A review of suicide prevention interventions in South Korea emphasizes the importance of culturally tailored SPI modifications to better assist researchers, mental health professionals, educators, parents, and policymakers (90).

Government recognize that fostering adolescent health leads to healthier adults who contribute to their communities while reducing costs associated with lost productivity and healthcare. Therefore, they support and invest in preventive mental health interventions. It is essential to develop comprehensive mental wellbeing programs that are effective in low- and middle-income countries (99).

A review of 27 studies recommended that Suicide prevention programs are most effective in enhancing awareness and helping skills, with comparatively smaller effects on suicide behavior, psychological wellness, and distress (100).

Integrating suicide prevention into national health primary care programs necessitates the design and implementation of evidence-based interventions both established and innovative (19). While comprehensive mental health care is crucial, it is equally imperative to have access to timely, responsive, and quality clinical care for early recognition and referral to counselors, for individuals showing suicidal behaviors (101).

The economic classification of countries, as defined by the World Bank, significantly influences healthcare investment and access to mental health services. Nations with a Gross National Income (GNI) of $1,005 or less are categorized as low-income, while those with a GNI between $1,006 and $3,955 fall into the lower-middle-income bracket (102). Many LMICs struggle to prioritize suicide prevention due to financial constraints, with only 10% possessing a national suicide prevention strategy (91). The availability of mental health professionals remains significantly lower in these regions compared to high-income countries, further exacerbating gaps in care. Given these limitations, suicide prevention efforts in LMICs must focus on developing cost-effective, culturally sensitive, and scalable interventions. Regional adaptation of strategies such as: community-based gatekeeper programs, integration of mental health into primary care, and the use of digital health solutions can enhance intervention sustainability (92).

### Policy and structural considerations in LMICs

A stark disparity exists between high- and low-income countries in suicide prevention efforts. Only 10% of LMICs have a national suicide prevention strategy, and mental health funding remains critically low (1). For instance, India allocates only $0.22 per capita to mental health care, while Pakistan dedicates a mere 0.4% of its healthcare budget to mental health services (103). Integrating suicide prevention into primary healthcare and adopting task-shifting strategies—such as gatekeeper training for non-specialist health workers, could help bridge this gap and expand access to care (104).

Moreover, in 35 LMICs, suicide attempts remain criminalized, deterring individuals from seeking help and exacerbating suicide-related mortality (105). Legislative reforms, coupled with public awareness campaigns, are necessary to shift societal perceptions of suicide from a criminal offense to a public health priority. Restricting access to lethal means—such as pesticides, firearms, and high-risk locations—has been identified as one of the most cost-effective suicide prevention strategies worldwide (21). In Bangladesh, the 2000 ban on WHO Class 1 pesticides led to a 37% drop in self-poisoning fatalities, preventing an estimated 35,000 suicides between 2001 and 2014 (106). Likewise, India’s 2011 endosulfan prohibition resulted in approximately 28,600 fewer pesticide-related suicides. In Sri Lanka, successive restrictions on Class 1 pesticides in the 1980s and 1990s, followed by additional bans (2008–2010), led to a 50% decline in pesticide suicides (2011–2015), particularly among young adults. Conversely, Iran reported a rise in aluminum phosphide poisonings after banning the substance in 2007 (107). The elimination of highly hazardous pesticides from agricultural use is expected to be a more effective suicide prevention strategy in rural Asia (108). While pesticide bans substantially reduced suicide rates, some declines were counterbalanced by increases in other suicide methods, emphasizing the need for comprehensive prevention strategies.

### Future implications

Contextually relevant and tailored interventions, addressing the unique needs of adolescents, coupled with ongoing evaluation, and collaboration among stakeholders, are essential for advancing suicide prevention efforts. Recommendations include the implementation of school-based mental health services, screening programs, and interventions targeting specific risk factors, such as substance use and interpersonal conflicts. Additionally, efforts are needed to destigmatize mental health issues, enhance access to support services at the school level, and to empower adolescents through resilience-building programs, for promoting mental wellbeing and for preventing suicide among this vulnerable population. To effectively prevent adolescent suicidality in LMICs, practitioners should collaborate across fields such as nursing, teaching, and counseling for capacity building to strengthen school-based support and improve access to care for at-risk youth (109). Mental health challenges significantly affect individuals, families, and communities, influencing societal aspects such as social and economic stability (104). Addressing this issue requires not only healthcare interventions but also school-based mental health programs, which play a vital role in promoting wellbeing and fostering a more equitable society (110).

Online interventions be utilized as a cost-effective and scalable means to support at-risk individuals. Additionally, the potential of passive smartphone monitoring as an inexpensive method for assessing risk warrants further investigation to establish its effectiveness (82). Limiting access to highly fatal suicide methods is a proven prevention approach especially in LMICs, as most survivors do not attempt again (82). Globally, restricting pesticides has significantly reduced suicide rates, particularly in rural areas of China, India, and Sri Lanka. Installing barriers, safety nets, surveillance, and conducting regular patrols at high-risk drowning sites can help prevent impulsive suicides (111).

Future suicide prevention efforts should focus on contextually relevant, culturally adapted, and sustainable interventions. Longitudinal studies are necessary to assess the long-term effectiveness of school-based, community-driven, and digital interventions in various socio-economic settings (112). The integration of digital innovations, including AI-driven mental health support, passive smartphone monitoring, and telehealth services, offers scalable and cost-effective solutions to bridge gaps in mental health care (113). Policy development remains crucial, with LMIC governments needing to prioritize national suicide prevention strategies and integrate mental health services into primary healthcare (1). Moreover, cultural adaptation is essential, as interventions must align with local beliefs, traditions, and economic conditions to maximize impact and community engagement (86). Improving mental health literacy through awareness campaigns is another key approach to reducing stigma and fostering proactive help-seeking behaviors (114). Additionally, limiting access to lethal means, such as pesticide bans, firearm control, and safety barriers at high-risk locations, has proven effective in reducing suicide rates, particularly in rural areas of China, India, and Sri Lanka (82, 111). Lastly, multisectoral collaboration among governments, educators, healthcare professionals, and policymakers is essential to develop sustainable, evidence-based strategies that comprehensively address adolescent suicide prevention.

### Strengths

This scoping review provides a robust synthesis of peer-reviewed literature on adolescent suicide prevention interventions, with a particular emphasis on LMICs. The review employed a rigorous methodology, utilizing systematic searches across major health databases to ensure a comprehensive evaluation of available interventions. One of the primary strengths of this study is its focus on suicide prevention strategies tailored to resource-limited settings, offering critical insights into culturally and economically viable approaches. Additionally, the review identified the use of validated outcome measures, ensuring reliability and accuracy in assessing intervention effectiveness. By incorporating an interdisciplinary perspective, the review highlights the crucial roles of educators, mental health professionals, policymakers, and researchers in designing and implementing suicide prevention programs. Moreover, the emphasis on school-based and community-driven interventions reinforces the importance of engaging adolescents directly in mental health promotion and suicide prevention efforts.

### Limitations

Despite its contributions, this review has several limitations. The exclusion of non-English studies may have resulted in the omission of valuable research, particularly from LMICs where regional languages dominate academic discourse. Additionally, while multiple databases were searched, the inclusion of gray literature, unpublished dissertations, and institutional reports could have further enriched the analysis. The limited scope of included studies and the 40% inclusion threshold may have restricted the dataset, potentially impacting the generalizability of findings. Furthermore, the review does not conduct direct assessments of intervention effectiveness, relying instead on secondary data, which may introduce biases related to study design and reporting. Given the vast socio-economic and cultural diversity among LMICs, the generalizability of findings across different regions remains a challenge, necessitating localized research to tailor interventions more effectively.

## Conclusion

This scoping review underscores the urgent need for comprehensive, evidence-based, and culturally sensitive suicide prevention interventions for adolescents, particularly in LMICs. The findings highlight the effectiveness of school-based programs, digital mental health interventions, and resilience-building initiatives in reducing adolescent suicide risk. While high-income countries have successfully implemented universal psychosocial programs, significant gaps remain in LMICs, where limited resources and a lack of mental health infrastructure pose considerable challenges. School-based interventions, including socio-emotional learning programs, peer support initiatives, and mental health literacy campaigns, have demonstrated promising results in enhancing adolescent wellbeing and reducing suicidality. Additionally, integrating suicide prevention strategies within primary healthcare, strengthening community support systems, and addressing structural barriers to mental health services are crucial steps toward reducing adolescent suicide rates globally. Future research should focus on longitudinal studies, culturally adapted interventions, and multi-sectoral collaboration to enhance suicide prevention efforts. By prioritizing sustainable and inclusive strategies, LMICs can make significant strides in improving adolescent mental health and mitigating the global burden of suicide.

## Data Availability

The original contributions presented in the study are included in the article/Supplementary material, further inquiries can be directed to the corresponding author/s.

## Footnotes

^1^ https://archive.org/details/osf-registrations-6w8d9-v1

## Appendix

**Table:**

Keyterms	Search engine	Hits
(“Prevention Program” OR “strategies” OR “intervention” OR “measures”) AND (“Adolescent” OR “Teenage” OR “school age”) AND (“Self-injurious behavior” OR “Self-injurious behavior” OR “suicide” OR “suicidal” OR “suicidality”) AND (“Suicidal Behaviors” OR (“mental wellbeing” OR “mental health” OR “psychological health”)) AND (“interventional” OR “Reviews”)	Pubmed	151
(program* OR intervention* OR Strategies OR preventive OR prevention OR prevent) AND (Self-destructive behavior OR Self-destructive behavior OR Self-injurious behavior OR Self-injurious behavior OR suicide OR suicidal OR suicidality OR suicidology OR “Suicide”[MeSH Terms])) AND (Adolescents OR School age OR teen OR youth) AND (mental wellbeing OR suicidal behaviors) AND (reviews OR RCT OR experimental)	Google Scholar	835
Adolescents AND Suicide AND Prevention	EBSCO	178
Suicide AND Adolescents AND Mental Health AND Suicide Prevention Intervention Prevention	PsychInfo	211
“Suicide”AND (“Mental Wellbeing” OR “Menal Health”) AND (“Prevention Program”OR “intervention”) AND (“Adolescent” OR “Teenage”)	Science Direct	196
